# Human-centered Design of a Health Recommender System for Orthopaedic Shoulder Treatment

**DOI:** 10.21203/rs.3.rs-4359437/v1

**Published:** 2024-05-21

**Authors:** Akanksha Singh, Benjamin Schooley, Jack Mobley, Patrick Mobley, Sydney Lindros, John M. Brooks, Sarah B. Floyd

**Affiliations:** University of Alabama at Birmingham; Ira A. Fulton College of Engineering, Brigham Young University; University of South Carolina School of Medicine Greenville; University of South Carolina; Clemson University; University of South Carolina; Clemson University

**Keywords:** human-centered design, health recommender system, human factors, orthopaedic treatment, personalized medicine, clinical decision support, proximal humerus fracture

## Abstract

**Background:**

Rich data on diverse patients and their treatments and outcomes within Electronic Health Record (EHR) systems can be used to generate real world evidence. A health recommender system (HRS) framework can be applied to a decision support system application to generate data summaries for similar patients during the clinical encounter to assist physicians and patients in making evidence-based shared treatment decisions.

**Objective:**

A human-centered design (HCD) process was used to develop a HRS for treatment decision support in orthopaedic medicine, the *Informatics Consult for Individualized Treatment (I-C-IT)*. We also evaluate the usability and utility of the system from the physician’s perspective, focusing on elements of utility and shared decision-making in orthopaedic medicine.

**Methods:**

The HCD process for I-C-IT included 6 steps across three phases of analysis, design, and evaluation. A team of informaticians and comparative effectiveness researchers directly engaged with orthopaedic surgeon subject matter experts in a collaborative I-C-IT prototype design process. Ten orthopaedic surgeons participated in a mixed methods evaluation of the I-C-IT prototype that was produced.

**Results:**

The HCD process resulted in a prototype system, I-C-IT, with 14 data visualization elements and a set of design principles crucial for HRS for decision support. The overall standard system usability scale (SUS) score for the I-C-IT Webapp prototype was 88.75 indicating high usability. In addition, utility questions addressing shared decision-making found that 90% of orthopaedic surgeon respondents either strongly agreed or agreed that I-C-IT would help them make data informed decisions with their patients.

**Conclusion:**

The HCD process produced an HRS prototype that is capable of supporting orthopaedic surgeons and patients in their information needs during clinical encounters. Future research should focus on refining I-C-IT by incorporating patient feedback in future iterative cycles of system design and evaluation.

## 1. Introduction

Evidence-based medicine has advanced significantly since its popularization in the 1990s, (1, 2) though many practice-specific gaps remain for the care of individual patients. It is well recognized that treatment effect heterogeneity erodes the value of average treatment effect estimates across groups of diverse patients (3–6), and a gap remains to generate evidence that applies to individual patients.(7–9) Rich data on diverse patients and their treatments and outcomes stored in Electronic Health Record (EHR) systems can be used to generate real world evidence suited for individual patients(10–15) Using historical patient data stored in EHR systems to assist with clinical decision-making was first conceptualized by researchers at Stanford and was referred to as an informatics consult service (ICS). (14–17) ICSs are designed to summarize historical data to help physicians make data-informed choices for current patients. However, the initial ICSs were focused on questions of prognosis not treatment choice, (17) and they were not fully automated, requiring a team of researchers and data scientists to field requests and return responses over a period of days. New computing advancements can be applied to fill the analytical gaps of early ICS systems and deliver real-time evidence to guide individual treatment decisions at the point of care.

Comparative effectiveness research (CER) has been used to generate evidence about treatment alternatives to inform treatment decision-making. Traditional CER approaches require researchers to generate average treatment effect estimates for a population subgroup or a “reference classes” based on a combination of measured baseline factors specified prior to estimation. (18–20) The need to define reference classes is a significant barrier to finding appropriate treatment effect estimates for individual patients. (20) Even with a small number of measured baseline factors, an individual patient could potentially be placed in an indefinite number of reference classes (19–21), and it is often unclear which reference class is best aligned to each patient. (19, 21–25) Here we start from the premise that physicians possess greater accumulated clinical wisdom on the patient factors that impact treatment effectiveness than researchers. (17, 26, 27) As such, physicians may be best positioned to define the appropriate reference class for a given patient in a specific setting and need a tool to generate CER-based evidence to summarize outcomes for similar patients under treatment alternatives.

Conceptually, Recommender Systems (RSs) provide an effective system foundation for delivering tailored evidence to guide individual decision making. RS are intelligent applications(28) that provide personalized information fit for the end user (29).A health recommender system (HRS) focuses on health-related information needs. The core HRS functionality of selectively presenting or “recommending” health data can be adapted to the need of presenting clinically appropriate reference class data summaries which closely match each patient being treated (30). Presenting personalized treatment evidence can enable engagement in shared decision-making, a priority goal in orthopaedic medicine.(31) Shared decision-making includes physicians and patients being informed about treatment options, evidence of the treatment effects on outcomes the patient can expect, and patient preferences across outcomes,(32) so that a patient may play an engaged role in their care. HRSs may offer a unique tool to facilitate shared decision-making in orthopaedic encounters, as HRSs can retrieve and present historical reference class patient data summaries that are matched to the individual patient. We use the HRS conceptual foundation to develop a clinical decision support system for orthopaedic medicine, the *Informatics Consult for Individualized Treatment* (I-C-IT, pronounced “I see it”).

The management of proximal humerus fracture (PHF) provides a useful clinical scenario for developing I-C-IT. There is significant variation in the management of PHF, (33–37) which is partially due to a lack of evidence to inform the choice between surgical options or conservative care. (38, 39) Meta-analyses across clinical trials seems to validate these concerns, suggesting the comparative effect of surgery versus conservative care is likely heterogenous across patients. (36) Surgical treatment is more costly, increases the risk of surgical complications and mortality, (40) and has not been shown in clinical studies to produce superior pain reduction or functional outcomes for many patients. (34, 35) Yet, surgery continues to be used at high rates in many regions of the country. (41, 42) It is unclear whether current surgery rates for many subsets of patients with PHF represent over or underutilization, with the key question not being whether surgery or conservative management is “the” best treatment, but rather which treatment is best suited for each patient. (43–49) Evidence on the comparative effects of alternative treatments that are appropriate for each patient (50) is needed to guide individual treatment choice.

In this study, we adopt a human-centered design (HCD) (51, 52)approach to identify design requirements and develop the HRS prototype - the I-C-IT for PHF treatment decision support. HCD prioritizes users’ needs and requirements during the system development process and is concerned with ways in which components of interactive systems can enhance human-system interaction. This work builds upon the original ICS concept(17, 53) to design and develop a HRS to perform dynamic searches and receive immediate comparative treatment and outcome data summaries to aid in shared treatment decision-making by physicians and patients at the point of care for PHF. In the final stage of the HCD process, we engaged ten orthopaedic surgeons in a mixed methods evaluation of the I-C-IT prototype that was produced.

## 2. Methods

**Figure 1:**
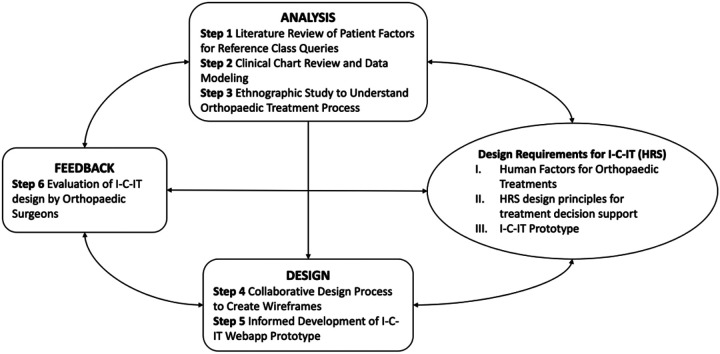
Human-Centered Design Process for Health Recommender System Instance – I-C-IT

The HCD process used to develop I-C-IT included 6 steps across three phases of design, analysis, and evaluation (Fig. 1). In addition to traditional steps involving process analysis (Step 3), and feedback through usability assessments (Step 6), we used a collaborative design approach where physician end-users were engaged in the design and evaluation process. Table 1 outlines the HCD phase, steps, and activities performed. A team of informaticians and comparative effectiveness researchers collaborated with input from orthopaedic surgeon subject matter experts. This study was reviewed and approved by the Institutional Review Board at Prisma Health (Protocol #1990879–1), and informed consent to participate was obtained from all study participants.

### Step 1. Literature Search of Patient Factors for Reference Class Queries

A *priori* the research team envisioned a system in which physicians apply accumulated wisdom to define the most appropriate reference class for a given patient. To achieve that envisioned design feature, a list of patient factors thought to modify treatment effectiveness was needed. A team of two researchers conducted a search of the literature to identify a list of patient factors associated with treatment effectiveness and treatment choice for PHF. A total of 10 articles were identified and reviewed from PubMed and Web of Sciences databases. Forty-five individual patient factors were identified and grouped into five domains: social independence, bone quality, patient mobility and activity level, surgical risk factors, fracture characteristics. These factors provided a comprehensive list of patient factors that could be used for reference class queries.

### Step 2. Clinical Chart Review and Data Modeling

A team of three comparative effectiveness researchers conducted a manual chart review in the EHR system to extract each of the individual patient factors identified from the literature review. Over 700 patient charts were reviewed. Structured and unstructured data fields were scanned to identify factors of interest. A regression analysis was used to identify the patient factors most relevant to treatment choice and relevant for reference class selection. Step 1 and 2 provided a refined list of patient factors which could be used for reference class identification.

### Step 3. Ethnographic Study to Understand the Orthopaedic Treatment Process

We conducted an ethnographic study (54, 55) to understand the process of treatment decision-making for orthopaedic surgeons and identify the decision points during an orthopaedic clinical visit, the design factors for HRS prototype, and elements for reference class for similar patient cohort selection. We shadowed two orthopaedic surgeons with different experience levels (< 15 years and 25 + years) in a large and busy clinical practice for a total of 11 hours encompassing 42 patient visits. The ethnographic study undertaken in this step helped identify the decision points, information elements involved in the treatment decisions, data evidence requirements and other design requirements to guide the design of desired functionality for the proposed I-C-IT system. The team intended to design a tool which would align with the current information seeking and decision-making process undertaken by physicians. This step elucidated key design features for the system, to maximize usability and utility for physicians. Based on observing the data needs, treatment decision-making process, and patient and physician discussions, we theorized the design elements to include data visualizations across three main areas 1) initial treatment utilization patterns and trends, 2) patient-reported outcome comparisons across treatment alternatives, and 3) 1-year healthcare utilization comparisons across treatment alternatives.

### Step 4. Collaborative Design Process to Create Wireframes

Next, we iteratively designed wireframes (i.e., visual mockups) for the I-C-IT prototype using the Adobe XD modeling tool. Two informaticians and three comparative effectiveness researchers conducted agile design sessions consisting of fifteen-day intervals inclusive of wireframe designing, gaining user feedback, prioritizing feedback across researchers, and creating revised designs. The collaborative design process spanned 6 months and produced roughly 15 iterations of I-C-IT. The resulting wireframes provided a vision for the desired system. This collaborative design process resulted in a system design that allows providers to define appropriate reference classes for a given patient using patient factor filters to query historical data. For that reference class, summarized data visualizations are presented for initial treatment utilization patterns and trends, patient-reported outcome and 1-year healthcare utilization comparisons across treatment groups.

At the end of the collaborative design process, 30-minute semi-structured interviews were conducted with four orthopaedic surgeons specializing in shoulder conditions. Physicians feedback on design elements was used to inform design enhancements and help refine overall design features in the I-C-IT prototype wireframes. Examples of enhancements that came from interviews included grouping patient demographic, fracture characteristics, and comorbidities together. Additionally, physicians desired an expanded PHF factor list to include the specific fracture location and amount of displacement, beyond just displacement and the number of fracture parts. On the third tab for healthcare utilization outcomes, one physician suggested adding a data element to report the percentage of initial conservatively managed patients that failed and converted to surgery. Smaller revisions included having treatment comparators in distinct colors to easily contrast treatment groups.

### Step 5. Informed Development of I-C-IT Webapp Prototype

**Figure 2:**
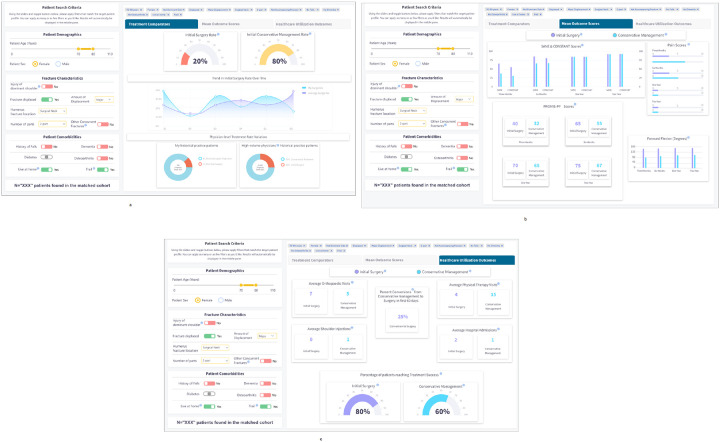
a. I-C-IT Webapp Prototype Screen 1 b. I-C-IT Webapp Prototype Screen 2 c. I-C-IT Webapp Prototype Screen 3

In this step, we developed the first high fidelity webapp prototype of I-C-IT. This prototype was built as a webapp driven by simulated data elements for 2,000 unique patients with PHF. It was designed to support two key future functions. The first function was integration into an EHR by incorporating a Java Script Notation (JSON) framework for patient search criteria and related outcome data. Second, a semantic web framework was applied using data elements in I-C-IT to provide a platform for AI-based algorithms to be implemented to support a background inference engine, while being integrated with EHR systems. Figure 2 shows images of the I-C-IT Webapp Prototype.

### Step 6. Evaluation of HRS I-C-IT Prototype Design

This phase of the HCD process focused on enhancing the I-C-IT prototype design by gathering feedback from 10 orthopaedic surgeons in a mixed methods study. We used a purposive, convenience sampling approach to recruit shoulder specialist orthopaedic surgeons from the regional practice network associated with our orthopaedic research group, the Center for Effectiveness Research in Orthopaedics (CERortho). For the purposes of this study we developed and conducted 60-minute qualitative interviews with each orthopaedic surgeon where we asked probing questions surrounding I-C-IT facilitators and barriers of use, and how I-C-IT might enhance their current practice in terms of engaging in shared decision-making (see full interview guide in the Appendix). We conducted a thematic analysis of interview data using the NVivo-12 software and applied a peer debriefing qualitative research method (56). Two interviewers independently reviewed the identified themes and reconciled the analysis to produce final evaluation results. At the end of the interview, we asked participants to complete a standard system usability scale (SUS) questionnaire (57) as well as a customized utility survey to evaluate several associated utility constructs including relevance of reference class filters, ease of navigation and understanding, interpretability of data representations, utility of the system in making evidence informed treatment decisions, and I-C-IT helpfulness in discussion with patients for shared decision-making. Table 2 provides information on participants that completed the interview and utility survey in Step 6.

## 3. Results

### Patient Demographics and Clinical Factors for Reference Class Selection

The goal of Step 1 and 2 of the HCD process was to generate a comprehensive list of patient factors that are relevant to treatment effectiveness and choice. The research team identified a total of 45 patient factors that were eventually minimized to 14 based on collaborative design feedback in Step 4. Patient factors selected for inclusion included patient age, gender, injury of the dominant shoulder, fracture displacement, amount of displacement, location(s) of the fracture, number of topographical parts involved, presence of concurrent fractures, patient frailty measured by a history of falls and a subjective frailty assessment, diabetes, dementia, osteoarthritis and whether the patient lives independently.

### Information Elements for I-C-IT

Decisions about what treatment and outcome information to present in the results tabs of I-C-IT were first envisioned during Step 3 and iteratively designed and evaluated in Step 4. Multiple rounds of design and feedback were undertaken, in which discussion centered on information presentation and visualization modification. The final version of I-C-IT (see Fig. 2a–c) contains 3 results tabs and a total of 14 data visualizations for the reference class. Table 3 provides the identified 14 informational elements for use in I-C-IT.

### Human-centered Design of I-C-IT

The aim of I-C-IT was to provide a clinical decision support tool that could stratify historical patient cohorts and summarize personalized treatment evidence to aid in clinical decision support. The design of the I-C-IT interface contains reference class search criteria on the left-hand side, while on the middle pane it contains various tabs presenting summarized data on treatment utilization, patient-reported outcome measure comparisons, and healthcare utilization for surgical and non-surgical treatment groups within the matched reference class criteria. The prototype was presented to orthopaedic surgeons in this study to assess whether the system captures and presents the design elements needed to support PHF treatment decision-making. Participants affirmed that the main key evidence and decision factors were represented. Future iterations will continue to refine desired system elements and functionality. Also revealed during the HCD process was a set of inductive HCD principles for HRS design to maximize usability and utility in a shared decision-making clinical context and setting. Table 4 represents the design features as they link to a human factor and design principle for HRS design.

### Mixed Method Evaluation of Usability and Utility of I-C-IT Webapp Prototype

**Figure 3:**
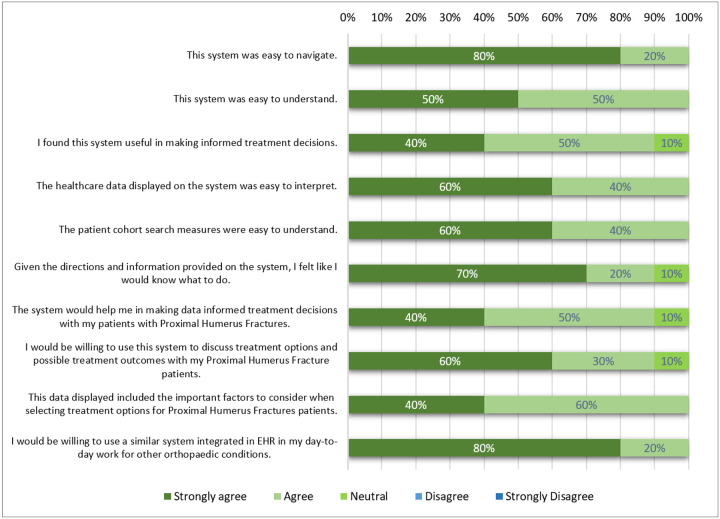
Results of Utility Survey

Usability was first addressed with individual orthopaedic surgeons in Step 4 and formally evaluated in Step 6. In the formal evaluation, the overall SUS score for the I-C-IT Webapp prototype was 88.75 indicating high usability (58). The utility survey results indicated overall positive feedback on the design of I-C-IT (Fig. 3). The response scores were on a 5-point Likert scale (5 = Strongly Agree to 1 = Strongly Disagree). Most orthopaedic surgeons responded between agree to strongly agree for all questions, indicating mostly strong agreement to questions on ease of navigation, ease of understanding, interpretability, utility of system in making data evidence informed treatment decisions, relevant cohort matching filters, helpfulness in discussion with patients for shared decision-making in orthopaedic treatments, appropriateness of data visualizations for PHFs, and willingness to use a similar system integrated in an EHR in their day to day treatment decisions for other orthopaedic conditions. Elements of shared decision-making were specifically addressed in Question 7, in which 90% of respondents either strongly agreed or agreed that I-C-IT would help them make data informed decisions with their patients (Fig. 3).

From the thematic analysis of the qualitative interview data in Step 6 of the HCD design process, a theme emerged surrounding how I-C-IT could enhance PHF treatment decision-making at point of care. Physicians confirmed the availability of tailored data elements and summaries within I-C-IT would facilitate richer conversations with patients about their preferences for outcomes of care and treatment choice. For instance, when asking one physician participant how he approached thinking about treatment decisions, he responded “*I think it’s comorbidities, mechanism, and then patient goals*.” This result signaled that beyond the clinical presentation of the patient (comorbidities and fracture characteristics), patient goals are highly relevant for treatment decision-making, and a summative presentation of outcomes would be helpful to share with patients as they weigh treatment choices.

Moreover, the amount of shoulder functionality desired by a patient at the end of treatment was described as one major factor in choosing a treatment option. One physician described an example of a functional goal for an older woman, “*The majority of the people who break their shoulder are older, and a lot of them are women, and the biggest problem is having difficulties with putting their bra on or combing their hair*.” Therefore, the presentation of shoulder range of motion scores in I-C-IT was highly valuable to have available for patient conversations when weighing treatment decisions. Additionally, many physicians consider pain reduction as a primary goal for most PHF patients, thus the availability of pain score outcomes by treatment group was highly desirable to physicians as it will be key for shared decision-making discussions. One physician participant noted how and when the I-C-IT tool could be used to educate and engage in shared decision making with their PHF patients on treatment options, “*When I talk to patients, I’d pull this up in a user-friendly way, I can spend 15 min with the patient coaching them in the office*.” All physician participants responded positively to the data elements that I-C-IT contained and the ways in which I-C-IT could be used to enhance clinical encounters with their patients.

## 4. Discussion

We describe the HCD approach used to develop I-C-IT, a prototype HRS designed to provide real-time CER-based evidence for the treatment of PHF. The long-term goal for I-C-IT is to improve physicians’ and patients’ ability to make evidence-based data-informed treatment decisions and select the treatment that is best aligned with treatment evidence and patient preferences. I-C-IT offers personalized evidence for the unique patient that is sitting in front of the physician, which provides more tailored information than systematic reviews or meta-analyses on groups of patients that may deviate from the current patient. Visualized data summaries on how similar patients have been treated brings a unique perspective to the clinical encounter that is not currently available and historical data summarized by I-C-IT may reveal previously concealed data relationships and patterns to help overcome common cognitive pitfalls. Further, this patient-specific data will enhance shared decision-making within orthopaedic encounters.

Also, I-C-IT delivers up-to-date evidence on rapidly evolving practice trends which is an advancement over the sluggish translation of peer-reviewed literature. In the future, each patient that is treated in clinical practice will be looped back into the I-C-IT data pool for future analysis through recommender system data pipelines, which creates an ongoing learning cycle for personalized visualizations. Thus, future patient treatment choice can be improved from the treatment of patients today. Through ongoing development, HRSs like I-C-IT may be useful for supplementing shared decision-making between physician and patients in making on-demand informatics consults during the clinical encounter at the point of care.

I-C-IT is similar in many ways to other decision support applications, though our system differs in a few meaningful ways. First, unlike other systems that depend solely upon computer-based algorithms to phenotype patients (59) or identify patient reference classes, we leverage a physician-driven reference class identification and data recommendation process to summarize information. Machine learning or artificial intelligence-based solutions use statistical methods to group patients by closeness or fit, but these methods are void of physician acumen and intuition. (11, 60, 61) Computer-based approaches lack transparency in how patient reference classes are defined and can lead to challenges in the ability to explain and interpret results ex post because physicians cannot clearly identify the defining factors used to group patients.(62)

Furthermore, there is hesitancy among patients and physicians to use computer-based recommender systems that lack the art of human element of medicine. (26, 27) Physicians possess accumulated clinical wisdom on the patient characteristics that are relevant when making treatment decisions. (17, 63, 64) Because of this unique knowledge, physicians may be better positioned to phenotype or define reference classes of patients. Furthermore, I-C-IT has the capability to track physician reference class search patterns over time and learn from physician behavior to create ‘user profiles’ that exist in most content-filtering recommender systems. This provides the best translation of physician expert knowledge into computerized patterns of patient cohort searches and their specific outcomes. The expertise that a physician “knows what to search for” can be used to train computerized models to automate searches around the index patient in future integrations with EHR systems.

### Limitations and Future Work

Our work is not without limitations. First, this study focuses on the development of a HRS for one orthopaedic condition. More studies are needed to expand the HCD methodology to the development of HRSs for other leading orthopaedic conditions such as atraumatic rotator cuff tears, spine injuries, or knee injuries. We envision that one day I-C-IT, or a future iteration on our HRS, will be embedded within the EHR so that it is readily available and accessible during the clinical encounter. Thus, since our target point of care environment for system use is the clinical encounter, we believe physicians will be the primary end-users. However, the data visualization summaries produced by I-C-IT would also be shared with patients during the clinical encounter, and thus a system evaluation from patients’ perspective is also *critical* to evaluate the impact of I-C-IT on the overall shared decision-making process at point of care. Finally, for the utility of the system to be thoroughly tested for its real-world application, I-C-IT must be integrated with a dynamic data source that periodically integrates new data evidence, such as the Orthopaedic Data Repository (OPDR) built at the Center for Effectiveness Research in Orthopaedics (CERortho), that contains multiple years of EHR data. The current I-C-IT prototype operates using a limited data set for the purpose of studying physician interactions with the interface. While this work provided significant foundational knowledge for a human centered orthopaedic HRS interface design, follow-on large-scale studies are needed to understand the possible clinical implications of the I-C-IT design and to assess impact of using HRSs such as I-C-IT for treatment decision support in clinical settings.

## 5 Conclusion

Through this study we described a HCD process for development of a prototype HRS refined for orthopaedic medicine. Historical EHR data holds important answers about how current and future patients might be treated. Informatics consult systems that enable dynamic learning from historical treatment decisions and outcomes hold great potential for improving the quality of care for all orthopaedic patients. However, studies such as the one presented in this paper are needed to build the foundational logic, decision factors, and human-computer interactions required for an HRS to have a positive clinical impact. The foundational design framework of I-C-IT offers a novel paradigm to accelerate learning from historical practice and associated outcomes for orthopaedic conditions.

## Figures and Tables

**Table 1:** Human-centered Design Process for I-C-IT

Phase	Steps	Activities
**ANALYSIS**	1. Literature Review of Patient Factors for Reference Class Queries	2 Comparative Effectiveness Researchers conducted literature review Compiled 45 patient characteristics relevant to PHF treatment effectiveness and treatment choice 4 months to complete
	2. Clinical Chart Review and Data Modeling	5 comparative effectiveness researchers Manual chart review of 750 patients Regression modeling, patient characteristics, and treatment choice 7 months to complete
	3. Ethnographic Study to Understand Orthopaedic Treatment Process	2 informatics researchers Observed 42 patient visits across 2 orthopedic surgeons Observations recorded about physician workflow, information sources utilized, and decision-making process 4 months to complete
**DESIGN**	4. Collaborative Design Process to Create Wireframes	2 software analysts, 2 informatics researchers, 1 comparative effectiveness researcher Agile design approach to create wireframes Biweekly scrum sessions for wireframe design for 6 months Design feedback collected in 3 iterations with 5 researchers and 4 orthopaedic surgeons
	5. Informed Development of I-C-IT Webapp Prototype	Testing performed by 2 Informaticians and 1 Comparative Effectiveness Researcher. Agile software development approach for 8 months to create prototype HRS.
**EVALUATE**	6. Evaluation of I-C-IT design by Orthopaedic Surgeons	2 Informaticians and 1 Comparative Effectiveness Researcher 10 semi-structured qualitative interviews with open-ended questions with orthopaedic surgeons. NVivo 12 analysis of the interviews’ transcripts and utility survey 4 months to complete

**Table 2:** Demographics of Physician Participants

Physician Characteristic	Number of Physicians
***Physician Age Group***	
25–34	4
35–44	3
45–54	1
55–64	2
**Gender**	
Male	9
Female	1
**Years of Experience**	
Under 5	5
5–10 Years	2
10–15 Years	1
15–20 Years	0
20–25 Years	0
25–30 Years	1
30 + Years	1

**Table 3:** Information Elements for I-C-IT

Themes in Information Elements	Information Elements	Timeframe
*Initial treatment utilization patterns and trends*	Initial treatment utilization rates (surgery vs. conservative management)	Overall historic aggregate
	Trends in initial surgery rate over time (shown by physician user and all other users)	Quarter-wise past calendar year
	Physician user historical treatment patterns	Overall historic aggregate
	High-volume physician treatment patterns	Overall historic aggregate
*Patient-reported outcome comparisons across treatment alternatives*	SANE & Constant Scores by treatment groups	3-months, 6-months, 1-year and 2-years
	Pain scores by treatment groups	3-months, 6-months, 1-year and 2-years
	PROMIS-PF Scores by treatment groups	3-months, 6-months, 1-year and 2-years
	Forward flexion by treatment groups	3-months, 6-months, 1-year and 2-years
*Healthcare utilization comparisons across treatment alternatives*	Orthopaedic visit utilization by treatment groups	Utilization average across 1-year period following index visit
	Shoulder injections by treatment groups	Utilization average across 1-year period following index visit
	Physical therapy visits by treatment groups	Utilization average across 1-year period following index visit
	Hospital admissions by treatment groups	Utilization average across 1-year period following index visit period
	Percentage of initial conservative management patients converted to surgery	Utilization average across 1-year period following index visit
	Percentage of patients reaching treatment success by treatment groups	Utilization average across 1-year period following index visit

**Table 4:** Human-centered Design Principles for HRS

I-C-IT Design Features	Human Factors for System Design	Design Principles
Familiar and utilitarian data elements for treatment decisions. Appropriate feedback on action. Accuracy and reliability in data analytics.	User trust in data evidence	Predictability
Physicians search history learning (content filtering) Similar search learning (content and collaborative filtering) Patient cohort learning (collaborative filtering) Patient’s treatment outcome preference- based risk benefit profiling of treatment options.	User preference integration	Personalization
Clear data cohort selection with data classifiers on top of visualization pane. Number of patients in selected cohort (N) dynamically displayed Information icons to define visualized concepts and metrics.	Increased interpretability and explainability of visual data elements	Transparency
Dynamic searches and cohort selection Instant visualization update Quick screen loading Capability for EHR integrated auto-search for the current patient’s record.	Instant screen updates and intuitive navigation	Latency
Context aware and personalized filtering of data cohort and data visualizations. Optional search elements for tailored cohort selection. User (physician) knowledge integration in data elements design. Intuitive mapping of element labels to real world practice.	Relevant data elements selection and display	Accuracy
Comprehensive visualization of data elements. Patient provider communication enhancement with visual data evidence for treatment options. EHR integration capability for increased utility. User-centered evaluation of design.	Reduced burden of gathering data evidence	Usefulness

## Data Availability

The datasets generated and/or analyzed during the current study are not publicly available due to the ongoing development of the clinical decision support tool but are available from the corresponding author on reasonable request.

## Abbreviations

EHR: Electronic Health Record
CER: Comparative effectiveness research
RS: Recommender system
HRS: Health recommender system
I-C-IT: Informatics Consult for Individualized Treatment
HCD: Human centered design
SUS: System usability scale
PHF: Proximal Humerus Fracture
PROM: Patient-Reported Outcome Measure
SANE: Single Assessment Numeric Evaluation
